# Optical System Based on Nafion Membrane for the Detection of Ammonia in Blood Serum Samples

**DOI:** 10.3390/bios12121079

**Published:** 2022-11-25

**Authors:** Elisabetta Pasqualotto, Erica Cretaio, Matteo Scaramuzza, Alessandro De Toni, Lara Franchin, Alessandro Paccagnella, Stefano Bonaldo

**Affiliations:** 1 ARC—Centro Ricerche Applicate s.r.l., 35132 Padova, Italy; 2 Up-Code s.r.l., 35132 Padova, Italy; 3 Department of Information Engineering, University of Padova, 35131 Padova, Italy

**Keywords:** absorbance, ammonia extraction, Berthelot reaction, optical biosensing, Nafion

## Abstract

The blood ammonia (NH_3_) level is one of the most important hepatic biomarkers for the diagnosis and monitoring of liver pathologies and infections. In this work, we developed an optimized optical biosensing method to extract and quantify the ammonia contained in complex-matrix samples emulating the blood serum. First, the approach was tested with solutions of phosphate-buffered saline (PBS) and ammonia chloride. Then, further trials were carried out with solutions of fetal bovine serum (FBS). The ammonia was extracted from the tested samples through a customized cell, and it was optically quantified by exploiting the indophenol reaction. The extraction cell included a cation-exchange membrane in Nafion, which was chemically pre-treated through cleaning procedures of sulfuric acid and hydrogen peroxide to keep a basic pH in the ammonia solution and to avoid contaminants in the membrane. From the NH_3_ solution, the indophenol reaction produced light-reactive indophenol dye molecules, which were used as colorimetric indicators. Through absorbance measurements of the indophenol dye solution at 670 nm wavelength, we were able to detect and quantify the ammonia level in the samples both with a spectrophotometer and a customized miniaturized read-out system, obtaining a detection limit of 0.029 µmol/mL.

## 1. Introduction

High concentrations of ammonia (NH_3_) in the blood may indicate severe pathologies and/or metabolic disturbances related to the liver [1,2,3,4]. Thus, the timely detection and the continuous monitoring of the ammonia levels in blood samples is fundamental to ensure the well-being of patients.

In the last decades, the development of highly sensitive and selective sensing methods for the detection of biomarkers has received ever-increasing interest [5,6]. The sensing methods of well-known biomarkers are typically performed in specialized biomolecular laboratories through the employment of bulky clinical analyzers. These procedures often result in being lengthy, expensive, and complex. On the other hand, the use of biosensors offers rapid, accurate, and on-site detection, performed even by non-specialized users [7]. Biosensors have attracted considerable research efforts in recent years such as in clinical diagnostics [8,9,10] and agri-food quality monitoring [11,12], allowing for the development of easily integrated systems to analyze biological samples directly at the point of care [13,14,15]. In particular, optical biosensors are considered valuable resources in clinical analysis since they allow for the detection and quantification of the presence of specific analytes in complex-matrix samples such as blood and urine, with high sensitivity, high rejection to external interferences, stability, and low noise [16,17,18].

Recent studies have proposed several sensors capable of detecting ammonia at relatively small volumes in air [19,20,21,22,23,24,25,26,27,28,29], focusing mostly on environmental application and the agri-food sector [20,21]. However, only a few works have proposed approaches that are specific for the point-of-care analysis of ammonia in blood samples. Furthermore, they are mainly based on electrical measurements [22,23,24], not considering the clinic environment, which prefers optical techniques. Few studies have reported on the detection of ammonia based on optical methods [25,26,27,28,29]. Most optical detection systems first include the separation of the ammonia from the blood, taking advantage of the ammonia volatility in alkaline conditions. Then, the ammonia is measured using different analytical methods, whose main results are summarized in Table 1. The most consolidated methods are based on colorimetric reactions such as ninhydrin [26], Nessler’s reagent [27], and the indophenol reaction [28,29]. Meanwhile, in [30], the ammonia level is quantified through the measurements in the pH variation. However, these proposed optical systems result in being quite complex and impractical nowadays, as they require lengthy and expensive setup procedures, and multiple chemical reactions that need to be performed in a controlled environment by trained personnel.

In this work, we propose an optimized sensing system, suitable for non-specialized users, to quantify the ammonia in blood-serum samples at the point of care. The system is composed of an extraction cell, based on a Nafion membrane, and by a miniaturized optical module to measure the absorbance. We introduced a new chemical treatment to the Nafion membrane with sulfuric acid and hydrogen peroxide to avoid undesired effects such as shifts induced by contaminants and by strong changes in the pH of the sample under test. The quantity of the extracted ammonia was then determined by applying indophenol, or the Berthelot reaction, which produces a light-reactive indophenol dye molecule with an absorbance peak at 670 nm. The proposed system was validated through a spectrophotometer with samples based on water, sodium acetate and blood serum.

## 2. Materials and Methods

### 2.1. Chemicals

All chemicals were of analytical grade and the water was ultrapure Milli-Q laboratory grade (MQ). Phosphate-buffered saline (PBS), hydrogen peroxide, sulfuric acid, ethylenediaminetetraacetic acid (EDTA), and fetal bovine serum (FBS) were purchased from Sigma Aldrich (St. Louis, MO, USA). The ammonia (NH_3_) was obtained from a solution of ammonia chloride (NH_4_Cl) in an alkaline solution of 1 M sodium acetate by applying an Ammonia Assay Kit (55R-1410, Fitzgerald, West Springfield, MA, USA), a non-enzymatic assay with a limit of detection (LOD) of 1 nmol of ammonia. The ammonia assay was based on a Berthelot reaction and the necessary phenolic reagents (R1 and R2) were included in the used kit. The rapid separation of plasma and the ammonia testing were not an issue in this application since we aimed to ultimately develop a completely closed system to avoid eventual evaporation problems.

### 2.2. Instruments and Setup

**Figure 1:**
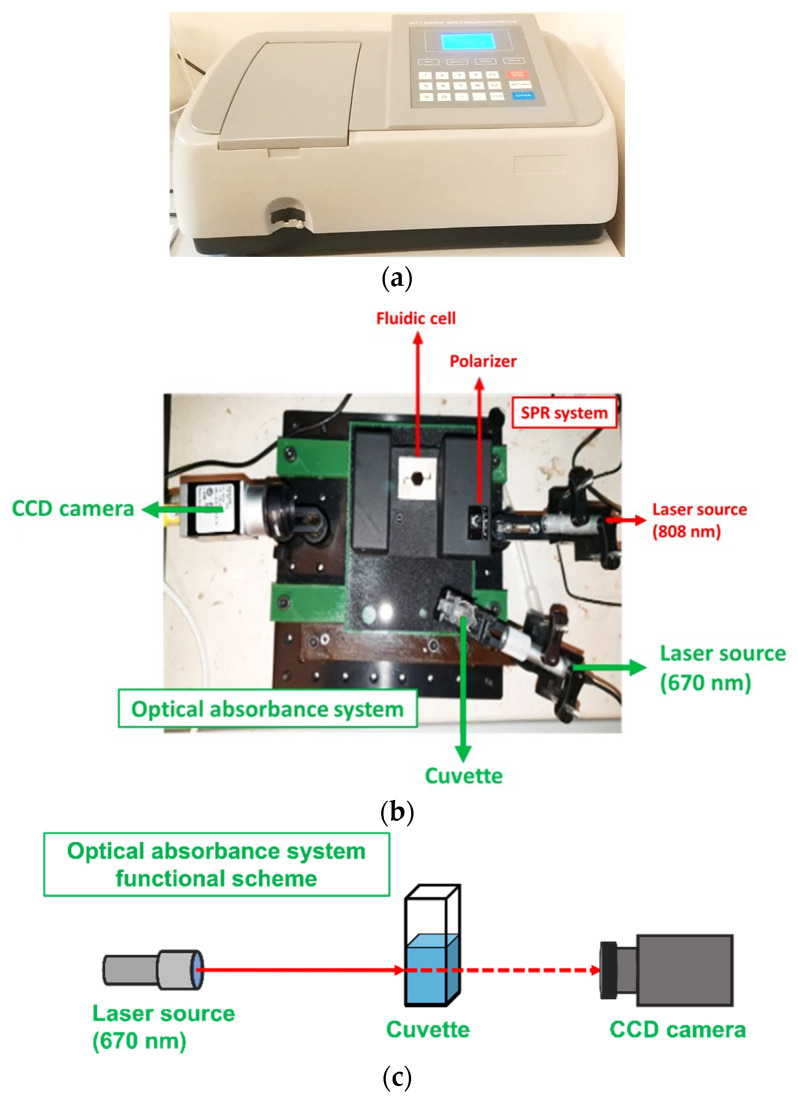
(**a**) The commercial spectrophotometer from Mapada Instruments, model UV-1600. (**b**) The proposed miniaturized optical system setup for the optical absorbance measurement, as evidenced in the green color. The image includes another SPR setup in the red color. (**c**) Functional scheme of the optical absorbance system.

Optical absorbance measurements were carried out with a spectrophotometer from Mapada Instruments, model UV-1600, in the wavelength range from 650 nm to 700 nm (see Figure 1a). The same optical measurements were also performed through a custom-made miniaturized optical system, as shown in Figure 1b. The system was composed of a 670 nm laser beam (Thorlabs, Newton, NJ, USA, CPS670F) and a cuvette mounted in a dedicated holder that assured the correct alignment between the light and the liquid inside the cuvette. The incident laser light was acquired by a CCD camera (Basler acA1300-60gc) and analyzed through a custom graphic-user interface. All of the data from the optical measurements were normalized to obtain an absorbance value between 0 and 1.

### 2.3. The Berthelot Reaction

The Ammonia Assay Kit 55R-1410 (Fitzgerald, West Springfield, MA, USA) was applied to carry out the ammonia detection with a colorimetric approach based on the Berthelot, or indophenol, reaction. In this chemical process, a basic pH solution of ammonia first reacts with a hypochlorite (OCl^−^) to obtain a monochloramine. Then, the compound reacts with two phenols (R1 and R2), forming an indophenol dye molecule, which is light-reactive at 670 nm and easily quantifiable by colorimetry using a plate reader or a spectrophotometer. Therefore, the concentration of NH_3_ can be determined by monitoring the absorbance of the final indophenol product of the Berthelot reaction. This reaction has been studied extensively for a variety of absorbing phenolic species and hypohalite sources. It has been reported in the literature that phenols in the Berthelot reaction can be replaced by several phenolic reagents such as sodium salicylate, which is non-toxic, reduces interferences, and improves color stability at slightly lower pH compared to those of phenols [31].

**Figure 2:**
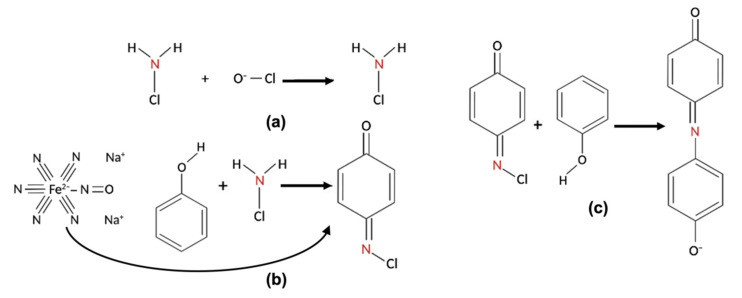
The steps of the Berthelot reaction. (**a**) The ammonia reacts with a hypochlorite, obtaining monochloramine; (**b**) Monochloramine reacts with R1, forming benzoquinone chlorimine, using sodium nitroferricyanide(III) dihydrate as a coupling reagent; and (**c**) benzoquinone chlorimine reacts with R2, forming the indophenol dye.

The three steps of the reaction are illustrated in Figure 2. In (a), the ammonia in the solution reacts with a hypochlorite (OCl^−^) to obtain monochloramine at basic pH. Then, in (b), the newly formed monochloramine reacts with the first phenol (R1) to form benzoquinone chlorimine, using sodium nitroferricyanide(III) dihydrate as a coupling reagent to increase the kinetics of the reaction. Finally, in (c), the product reacts with the second phenol (R2) to form the indophenol dye and, after a final incubation of 30 min at 37 °C, the absorbance of the dye molecule in the solution is measured at 670 nm.

## 3. Design and Treatments

### 3.1. Extraction Cell Design

**Figure 3:**
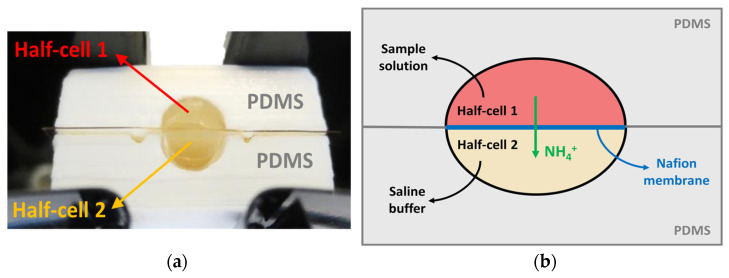
(**a**) Extraction cell setup. (**b**) Functional scheme of the extraction cell. The cell sections are made in PDMS (grey). The two wells are separated by the Nafion membrane (blue), through which the ammonium ions are extracted (green). The half-cell 1 (red) was filled with the sample and the half-cell 2 (yellow) was filled with 1 M sodium acetate.

To perform the analysis, limiting the possibility of interferents, the blood samples need to be filtered so that NH_3_ can be extracted and treated to obtain a light-reactive dye. To reach this aim, a 3D-printed extraction cell based on a cationic-exchange membrane in Nafion was designed (Figure 3). The Nafion membrane was previously subjected to a pre-treatment based on sulfuric acid to eliminate eventual impurities to avoid potential contaminations that may influence the basic pH of the solution.

The extraction cell was composed of two halves (1 and 2), separated by the membrane: the half cockpit was filled with 150 µL of the sample and the other half was filled with 150 µL of 1 M sodium acetate. To avoid fluid leakage during analysis, a 10% polydimethylsiloxane (PDMS) layer (0.1 mm^2^) was placed on the two sections of the cell. In these conditions, the NH_3_ extraction was obtained by incubating the cell for 30 min at 25 °C to allow for the complete passage of the ammonia from the sample to the second half-cell. Finally, a 100 µL drop of the extracted solution was withdrawn and put into a cuvette, where 80 µL of R1 and 40 µL of R2 were added to perform the assay, as previously described in Section 2.3.

### 3.2. Nafion Membrane as Ion-Exchange Barrier

Nafion is a tetrafluoroethylene sulfur copolymer used as a protonic conductor for protonic membrane cells (PEMs) as fuel for its excellent thermal and mechanical stability. When cast into films, the sulfonated block aggregates into long-range pores of sulfones surrounded by a matrix of fluoropolymer. The pores usually have a size of 1–4 nm and are highly negatively charged due to the sulfonic acid groups [32]. These pores allow for the rapid diffusion of hydroxyl containing molecules and cations while inhibiting the diffusion of anions and macromolecules.

**Figure 4:**
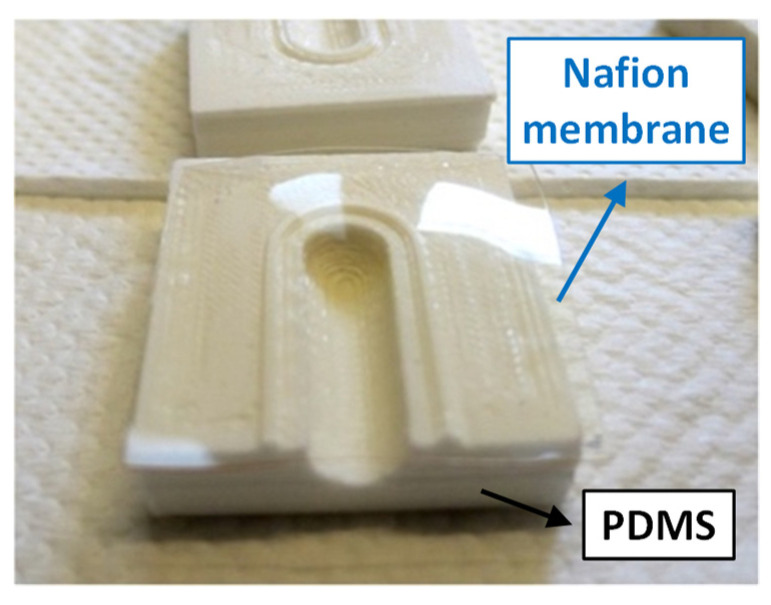
The Nafion membrane (highlighted by the blue arrow) on one half of the PDMS extraction cell.

In the extraction cell, the Nafion membrane was used as an ion-exchange barrier to retrieve the ammonia from the sample (Figure 4). A sodium ion from the sodium acetate well flows into the first well solution, trading places with an ammonium ion, effectively extracting the ammonia from the sample. The dimension of the membrane was 2.25 cm^2^, which is sufficiently large to avoid any contamination between the two wells.

#### Chemical Pre-Treatment of Nafion

Nafion is a very strong acid with a pKa of −6 due to the combination of fluorinated backbone, groups of sulfonic acid, and the stabilizing effect of the Nafion polymer matrix. Since the Berthelot reaction needs to occur in a highly basic environment, the sodium acetate solution pH was chosen to be 11, in order to mitigate the acidification effect due to the contact with the membrane. Furthermore, before assembling the extraction cell, Nafion was thoughtfully treated with chemical cleaning procedures to eliminate eventual contaminants that could lower the pH of the final solution and interfere with the reaction. The Nafion membrane was incubated initially in a 3% solution of hydrogen peroxide for one hour, then soaked in water for two hours. Afterward, the Nafion was left in a solution of 0.5 M sulfuric acid for one hour. Finally, the previous incubations were repeated first with water and then with hydrogen peroxide. During the whole process, the membrane and the solutions were kept at the temperature of 80 °C. The newly treated membrane was then stored in 1 mM EDTA to avoid desiccation before utilization.

The proposed Nafion treatment can also be a promising starting point for membrane cleaning after ammonia extraction, in order to reuse the cell after one measurement. The method needs to be further improved by possibly adding other chemical treatments to clean all of the residual contaminants on the Nafion, ensuring the effectiveness of an ulterior filtration using the same cell.

## 4. Results and Discussion

### 4.1. Calibration of the Ammonia Detection in Water-Based Solutions

**Figure 5:**
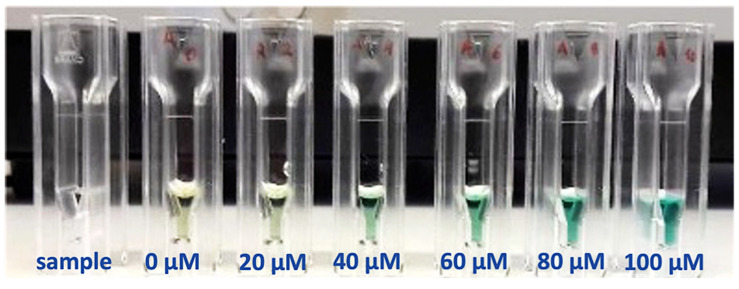
Color gradient obtained at different ammonia concentrations for the Berthelot reaction. The transparent sample solution assumes a gradient from light yellow (0 µM) to deep light aquamarine (100 µM) after the reaction by increasing the concentration. The quantity of ammonia present in the solutions can be determined by monitoring the absorbance of the dye molecule at λ = 670 nm.

The commercial Ammonia Assay Kit 55R-1410 was tested initially in aqueous solutions at different concentrations of ammonia to evaluate its performance and obtain a preliminary calibration curve. The spiked samples were prepared starting from a solution of 1 mM ammonium chloride in PBS, generating the concentrations of ammonia reported in Table 2. A volume of 100 µL of each sample was put in a reduced volume cuvette in UV-grade plastic. Then, reagents R1 and R2 were added, and the solution was incubated for 30 min at 37 °C to perform the Berthelot reaction. At the end of the incubation time, the transparent sample solutions assumed a gradient aquamarine coloration proportional to the ammonia concentration (Figure 5).

The optical measurements of the final solutions were carried out through a spectrophotometer, taking the absorbance value of the 0 µM solution as the baseline. The acquisition was performed in a wider range of wavelengths as the results showed that the maximum absorbance peak was not perfectly set at 670 nm, but it varied slightly as a function of concentration in a range between 650 nm and 700 nm. Nevertheless, the overall absorbance of the solutions was evaluated at 670 nm, as suggested by the manufacturers of the kit.

**Figure 6:**
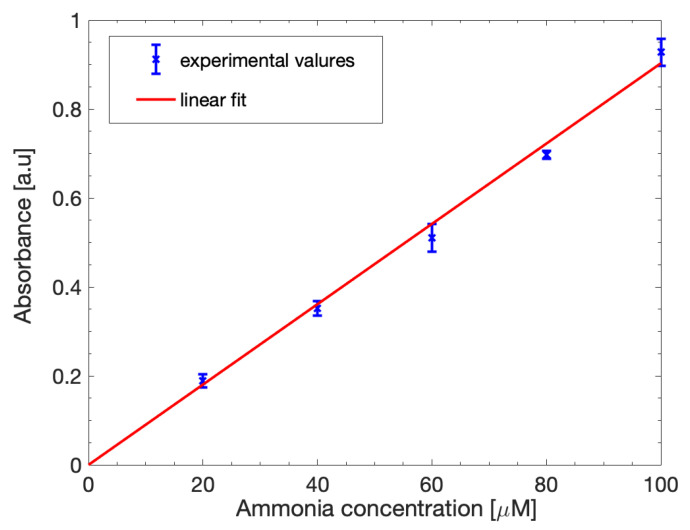
Ammonia calibration curve in ideal H_2_O solutions. The experimental absorbance data (blue points) were fitted with a linear equation (red line).

Figure 6 shows the obtained calibration curve. The curve can be fitted by a linear model (1) as expected. The slope of the linear fit (parameter *p*_1_) was estimated as 0.00903 with a 95% confidence interval and the correlation coefficient was 0.9965.

(1) $$f\left(x\right)=p_{1}\cdot x,$$

The assay was repeated multiple times at the same concentrations to assess its repeatability, obtaining just a slight variation of 6.4% between the tests, showing the consistency of the results.

### 4.2. Calibration of the Ammonia Detection in Sodium Acetate Solutions

The alkaline solution in the second well of the extraction cell is necessary for the ammonium ion exchange from the sample through the Nafion membrane. To assess its influence on the Berthelot reaction and on the ammonia absorbance linearity, a calibration curve was identified using an alkaline solution of sodium acetate (CH_3_COONa) to prepare the spiked samples, confirming the system linearity, which allows one to avoid false positive or false negative results.

The ammonia samples were prepared at a concentration of 20 µM, 60 µM, and 100 µM, starting from a solution of 1 mM ammonium chloride in PBS using 1 M sodium acetate instead of water as the diluent. A volume of 100 µL of each sample was put in a reduced volume cuvette in UV-grade plastic before adding reagents R1 and R2 and incubating for 30 min at 37 °C to obtain the indophenol dye molecule. At the end of the incubation time, the alkaline solutions assumed a gradient coloration proportional to the ammonia concentration similar to the aqueous solutions.

The optical measurements were carried out at the same previously described conditions, evaluating the absorbance peaks in a range of wavelengths between 650 nm and 700 nm.

**Figure 7:**
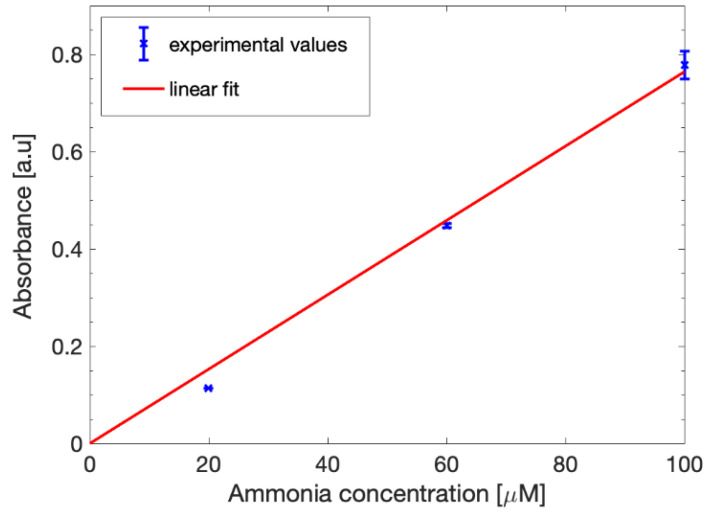
Ammonia calibration curve in 1 M sodium acetate. The experimental absorbance data (blue points) were fitted with a linear equation (red line).

The calibration curve is shown in Figure 7. While the measured values were slightly lower than the aqueous solutions, the curve could still be fitted with the linear model defined in (1), as expected. The slope was retrieved from the parameter *p*_1_ and was estimated as 0.0764 with a 95% confidence interval and the correlation coefficient was 0.9914. From these results, it is clear that the alkaline solution influence is very limited and does not prevent the proper occurring of the Berthelot reaction, allowing us to effectively detect and quantify the ammonia present in the samples.

### 4.3. Influence of the Extraction Cell on the Ammonia Detection Effectiveness

After the calibration of the system in alkaline solution, the test was repeated using the extraction cell, evaluating its influence on the absorbance measurements. A sample of 150 µL of 1 mM ammonium chloride in PBS was poured into the first well of the cell, while 150 µL of 1 M sodium acetate was inserted into the second well. The cell was then sealed, and the two solutions were left incubating for 30 min at 25 °C, permitting the ammonium ions to flow toward the sodium acetate solution. Afterward, a 100 µL drop was withdrawn from the second well and put into a cuvette, adding 80 µL of R1 and 40 µL of R2, and incubating the final solution for 30 min at 37 °C, as required from the reaction protocol described in Section 2.3. A sample solution without ammonia was used as the negative control.

**Figure 8:**
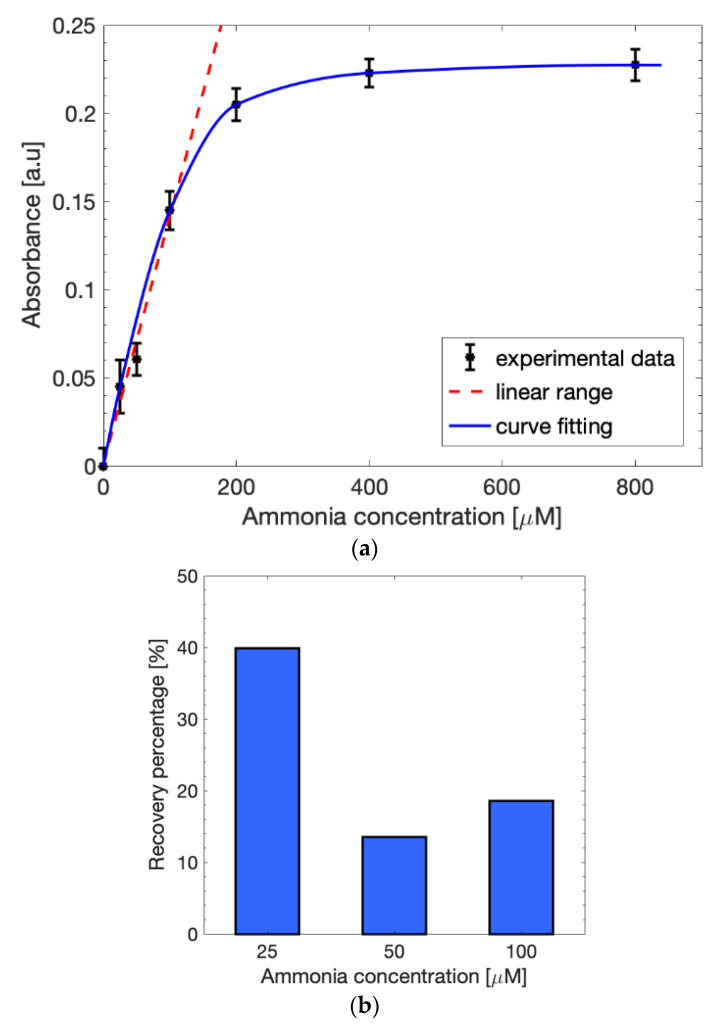
(**a**) Calibration curve in 1 M sodium acetate after the use of the extraction cell fitted with the Lambert–Beer law (blue line). The linearity (red dashed line) is maintained in the concentration range between 0 µM and 100 µM. (**b**) Recovery percentage in the linear range after the use of the extraction cell.

Six different solutions of ammonium chloride in sodium acetate, with an ammonia concentration range varying between 0 µM and 800 µM, were tested to identify the absorbance detection sensitivity of the method at 670 nm with a spectrophotometer (Figure 8a). The curve, fitted with the Lambert–Beer law (blue line), showed a linear behavior (red dashed line) in the interval between 0 µM and 100 µM, which is an optimal range since the normal blood ammonia level is usually between 11 µM and 32 µM and values above 50 µM are considered toxic for the human body. The linear range could be fitted with (1) and the obtained slope (*p*_1_) was estimated as 0.00154. The recovery percentage of the spiked samples in the linearity range was calculated to better understand the effects the extraction cell on the measurements. Figure 8b shows an evident reduction in the absorbance values obtained with the extraction cell with respect to the results without any solution filtering, as expected. The higher recovery percentage was obtained for 25 µM (~40%), while 50 µM and 100 µM led to lower values (~15%). This may be due to the Nafion membrane in the cell, which influences the flow of the ammonium ions during the 30-min incubation needed for the extraction. Nevertheless, the presence of ammonia was easily detectable by considering the final application concentration range.

**Figure 9:**
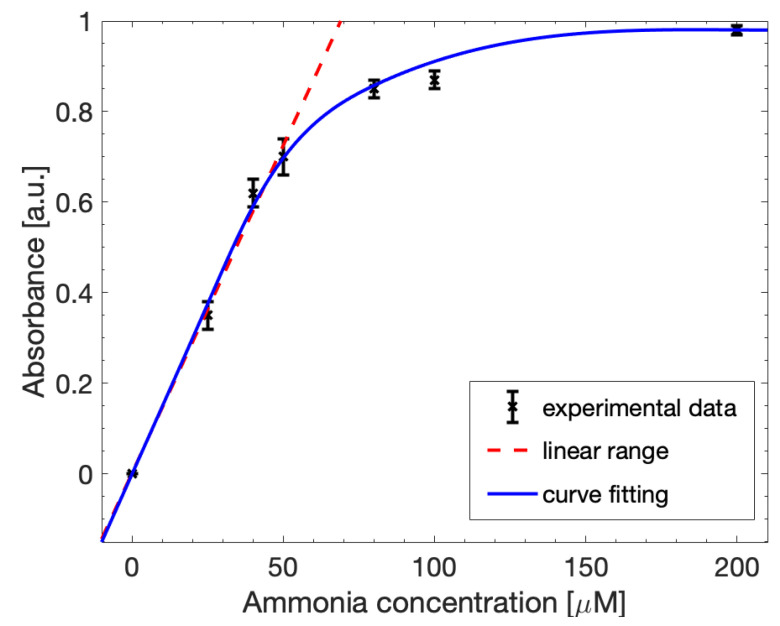
Calibration curve for ammonia detection of the SNIPE system fitted with the Lambert–Beer law (red line). The linearity (red dashed line) was maintained in a concentration range between 0 µM and 50 µM. The limit of detection was 0.028252 µmol/mL.

The same optical measurements were also performed through the custom-made miniaturized optical system reported in Section 2.2. Seven different concentrations of ammonia were analyzed in a range from 0 µM to 200 µM, setting the CCD camera exposure time at 4500 µs. Figure 9 shows the detection curve obtained for these concentrations. The detection limit was very low at about 0.028 µmol/mL, estimated considering 3*SD, where SD is the standard deviation of 0 µM ammonia solution. The linear range, between 0 µM and 50 µM, resulted in being lower than the one obtained through the spectrophotometer, and can still be fitted through (1), with a slope value of 0.01535.

The miniaturized optical system showed a good detection capability and a similar behavior to the spectrophotometer. While the linearity range of the instrument results was quite low, a sample dilution was sufficient to reach a suitable ammonia concentration to perform the detection. Hence, the customized optical system was capable of detecting the presence of ammonia in an ideal solution, yielding promising results for the analysis of more complex blood-based samples.

### 4.4. Influence of Complex-Matrix Samples on the Ammonia Detection Effectiveness

To evaluate the effect of a more complex matrix on the detection capability of the approach, a test in blood-like samples was performed using the extraction cell. The samples were prepared by replacing the PBS with fetal bovine serum (FBS) to obtain a more complex matrix simulating the serous component of the blood, as reported in Table 3. Control solutions were prepared using PBS-based samples.

**Figure 10:**
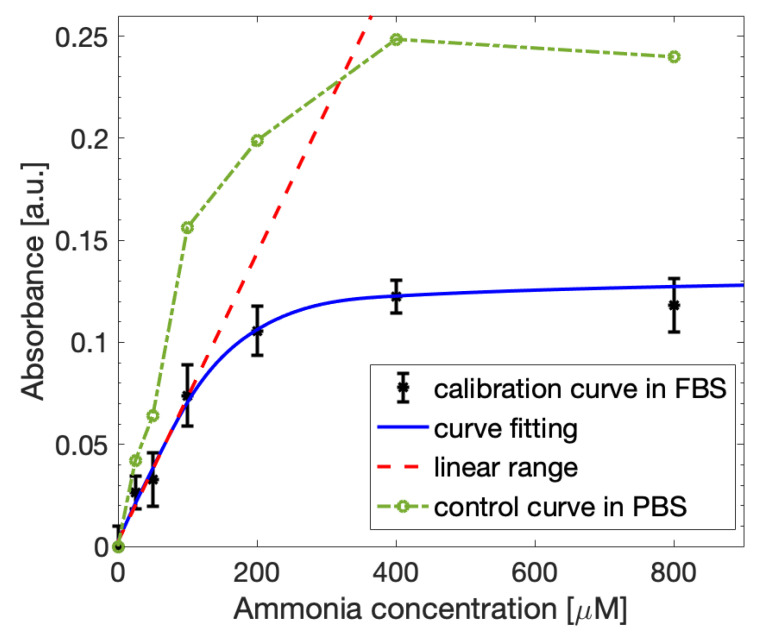
Ammonia calibration curve obtained with the spectrophotometer for FBS-based samples fitted with the Lambert-Beer law (blue line). The linearity (red dashed line) is maintained in the concentration range between 0 µM and 100 µM. The curve is compared with the calibration curve obtained for control PBS-based samples (green line).

The ammonia extraction from the blood-like samples and the control solutions was performed as described in Section 3.1. After the incubation procedures and occurring of the Berthelot reaction to obtain the dye molecule, the absorbance measurements were carried out at 670 nm with a spectrophotometer to outline the calibration curve for the FBS-based samples (Figure 10). The experimental results obtained for the blood-like solutions (black points) showed lower absorbance values than the controls (green points), meaning that the presence of a more complex matrix partially affects the quantity of extracted ammonia. Nevertheless, the curve assumes a behavior similar to the PBS-based control, following the Lambert–Beer law, with a linear interval between 0 µM and 100 µM and a slope value of 0.00071, which is once again suitable to the application to blood ammonia detection. The limit of detection was 0.029 µmol/mL, similar to the LOD retrieved for PBS. Moreover, the LOD of the sensor result was satisfactory with respect to other methods present in the literature [21,22,23,26], as the reported LOD values were in a range between 0.004 µmol/mL and 0.088 µmol/mL. Therefore, the proposed method results were capable of detecting the ammonia present in more complex solutions, emulating thee blood-based samples, with a performance in the linear range similar to the ideal case.

## 5. Conclusions

In this work, we proposed an optimized biosensing method to detect the extracted ammonia in complex-matrix samples, aiming toward a final application on hepatic patients. The system has been tested with ammonium chloride solutions in order to evaluate its detection capability in ideal and blood-like conditions. We demonstrated that an extraction cell, based on a Nafion membrane and an alkaline solution, is an effective method to retrieve the ammonia from the samples. Moreover, we optimized the extraction process by introducing a chemical pre-treatment of the membrane, based on sulfuric acid and hydrogen peroxide, to avoid low pH values and any contaminant in the final solution. After the extraction, a light-reactive molecule for the absorbance measurement was obtained from the Berthelot reaction of the ammonia. The detection method was tested with both a spectrophotometer and with a customized miniaturized optical system, yielding good performances with an estimated limit of detection of 0.029 µmol/mL. The approach was also successfully tested with complex-matrix samples to emulate the blood serum component, obtaining similar performances. Future works will be aimed toward testing the ammonia extraction approach with blood-based samples in order to evaluate the detection capability of the method with a more complex biological sample matrix.

## Figures and Tables

**Table 1:** Summary of the results of the optical ammonia sensors.

Detection Method	Linear Detection Range	LOD	Ref.
Chemiresistive	2 mM–8 mM	2 mM	[20]
ZnO/NiO nanocomposites	N/A	58 µM	[21]
Gas-phase sensor	25 µM–100 µM	4 µM	[22]
Impedimetric	25 µM–200 µM	12 µM	[23]
Ninhydrine reaction	200 µM–1400 µM	88 µM	[26]
Berthelot’s reaction	0.1 µM–10 µM	N/A	[28]

**Table 2:** Water-based solution compositions for each ammonia concentration.

Solution Composition	NH_3_ 0 µM	NH_3_ 20 µM	NH_3_ 40 µM	NH_3_ 60 µM	NH_3_ 80 µM	NH_3_ 100 µM
NH_4_Cl 1 mM [μL]	0	2	4	6	8	10
H_2_O [μL]	100	98	96	94	92	90
R1 [μL]	80	80	80	80	80	80
R2 [μL]	40	40	40	40	40	40
Total [μL]	220	220	220	220	220	220

**Table 3:** Solution compositions based on fetal bovine serum (FBS) for each ammonia concentration.

Solution Composition	NH_3_ 0 µM	NH_3_ 25 µM	NH_3_ 50 µM	NH_3_ 100 µM	NH_3_ 200 µM	NH_3_ 400 µM	NH_3_ 800 µM
NH_3_ 1 mM (μL)	0	2.5	5	10	0	0	0
NH_3_ 10 mM (μL)	0	0	0	0	2	4	8
FBS (µL)	90	90	90	90	90	90	90
H_2_O (μL)	10	7.5	5	0	8	6	2
Total (μL)	100	100	100	100	100	100	100

## Data Availability

Not applicable.
